# Similar mortality rates in hip fracture patients over the past 31 years: A systematic review of RCTs

**DOI:** 10.3109/17453674.2013.878831

**Published:** 2014-02-25

**Authors:** Simran Mundi, Bharadwaj Pindiprolu, Nicole Simunovic, Mohit Bhandari

**Affiliations:** Division of Orthopaedic Surgery,McMaster University, Hamilton, Ontario,Canada.

## Abstract

**Background:**

Over 320,000 hip fractures occur in North America each year and they are associated with a mortality rate ranging from 14% to 36% within 1 year of surgery. We assessed whether mortality and reoperation rates have improved in hip fracture patients over the past 31 years.

**Methods:**

3 electronic databases were searched for randomized controlled trials on hip fracture management, published between 1950 and 2013. Articles that assessed the surgical treatment of intertrochanteric or femoral neck fractures and measured mortality and/or reoperation rates were obtained. We analyzed overall mortality and reoperation rates, as well as mortality rates by fracture type, comparing mean values in different decades. Our primary outcome was the change in 1-year postoperative mortality.

**Results:**

70 trials published between 1981 and 2012 were included in the review. Overall, the mean 1-year mortality rate changed from 24% in the 1980s to 23% in the 1990s, and to 21% after 1999 (p = 0.7). 1-year mean mortality rates for intertrochanteric fractures diminished from 34% to 23% in studies published before 2000 and after 1999 (p = 0.005). Mean mortality rates for femoral neck fractures were similar over time (∼20%). Reoperation rates were also similar over time.

**Interpretation:**

We found similar mortality and reoperation rates in surgically treated hip fracture patients over time, with the exception of decreasing mortality rates in patients with intertrochanteric fractures.

Hip fractures pose a substantial challenge to both patients and healthcare systems worldwide, as the incidence of this condition continues to rise while the associated morbidity and mortality persist (Cooper et al. 1992, Schemitsch and Bhandari 2009). At present, over 320,000 hip fractures occur in North America alone each year, and this number is expected to rise to 580,000 by 2040 with healthcare costs exceeding 10 billion dollars (Cooper et al. 1992, Schemitsch and Bhandari 2009). Globally, it is projected that over 6 million hip fractures will occur annually by the year 2050 (Cummings et al. 1990).

Hip fractures in the elderly lead to functional decline and a diminished quality of life. Furthermore, these fractures are associated with an in-hospital mortality rate of 7–14%, reaching 14–36% within 1 year of surgery (Weller et al. 2005, Bottle and Aylin 2006, Schemitsch and Bhandari 2009, Simunovic et al. 2011, Murphy et al. 2013). Hip fractures are also complicated by a 0–49% need for revision surgery, which is influenced heavily by fracture characteristics and surgical interventions (Murphy et al. 2013).

Given the interest in identifying strategies to improve outcomes in hip fractures over the last 5 decades, we would anticipate significant improvements in major outcomes, as has been the case with mortality after myocardial infarction and stroke (Carandang et al. 2006, Setoguchi et al. 2008). For instance, mortality rates from coronary artery disease in the USA decreased by over 40% within 20 years (1980 to 2000), which prevented 340,000 deaths in the year 2000. Nearly half of this reduction in mortality has been attributed to the introduction of “evidence-based medical therapies” (Ford et al. 2007).

It remains unclear whether mortality after hip fractures has seen similar improvements; few analyses of trends in mortality rates have been published, and those that have appeared have shown conflicting results (Haleem et al. 2008, Brauer et al. 2009). We therefore assessed whether mortality rates for hip fractures have improved over the past 50 years, based on the best available evidence from the literature. We also analyzed trends in reoperation rates.

## Methods

We systematically reviewed the literature to identify clinical trials evaluating the surgical management of hip fractures. We used components of the PRISMA 2009 checklist that were applicable to our review.

### Assessment of eligibility for the study

The criteria for inclusion in the study were articles (1) published between January 1950 and January 2013, (2) published in the English language, (3) with a randomized controlled trial study design, (4) with surgical treatment of intertrochanteric or femoral neck fractures, and (4) with mortality and/or reoperation rates reported either as a primary or a secondary outcome.

### Search strategy

2 reviewers (SM and BP) searched the following 3 databases: MEDLINE, EMBASE, and PubMed. The search strategy combined the following terms: “hip fracture*”, AND “mortality*”, OR “reoperation*”.

### Study selection and data collection

Both reviewers screened the titles and abstracts of all studies identified in the search, and full-text articles were obtained for all trials deemed potentially eligible. The full-text articles were assessed for final inclusion using an eligibility screening document that was based on pre-specified inclusion and exclusion criteria. All studies were screened independently by both reviewers. Any disagreement about inclusion of articles in the study was resolved through a consensus discussion.

A data abstraction form was used to retrieve data regarding study characteristics and outcomes for all the trials included. Both reviewers independently extracted the data and further validated the extracted data through consensus discussion. The data abstracted included: journal, year of publication, demographic data (mean patient age, geographic location etc.), type of fracture, intervention, overall mortality rate at all reported time points (as a percentage), and reoperation rates at all reported time points (as a percentage).

If more than 1 publication from the same study was found, all versions were considered (to maximize data extraction) and the primary publication was identified along with the secondary references. Preliminary results were also included for similar reasons.

### Data analysis

We used descriptive statistics, consisting of frequencies and percentages, to report and compare outcomes. Our primary outcome of interest was the change in 1-year postoperative mortality rate (percentage) over time. An average mortality rate was calculated from all the studies published within a defined time period. In calculating the average mortality for each time period, the mortality rate from each trial was weighted by the sample size of that trial. We established a minimum cutoff of 3 primary mortality rates to proceed with an aggregate calculation of a mean mortality rate. We compared mean mortality rates in different decades (the 1980s, the 1990s, and after 1999) if enough mortality data were available, otherwise we compared mortality rates across eras (pre-2000 and post-1999). Subgroup analysis was carried out by fracture type. To determine the statistical significance of any detected changes in mortality rates over time, we used the Brown-Forsythe variant of the analysis of variance (ANOVA) test for comparisons between different decades. Student’s t-test was used for comparisons between different eras. We tested equality of variance using Levene’s test for equality of variances. Statistical significance was set at p = 0.05 and all tests were 2-tailed. An equivalent method of analysis was used for overall reoperation rates over time.

For articles reporting mortality and reoperation rates as a percentage, the value reported was used directly. For articles not reporting a percentage, mortality rates were calculated based on the number of patients rather than the number of hips. If follow-up was reported as a range, then rates were determined based on the endpoint of the follow-up.

## Results

Our search returned 850 articles for title and abstract review. Of these, 94 were retrieved for full-text review as they met our inclusion criteria or required further evaluation to assess eligibility. 20 of these articles were excluded because of not meeting the eligibility criteria (n = 2), because of being duplicate publications (n = 10), or from inability to access the full text through our institution (n = 8). Our final review included 70 trials, with 4 additional long-term follow-ups of previously published RCTs, giving 74 publications.

### Study characteristics

The 70 trials included in our final review were published between the years 1981–2012, representing a period of 31 years. Most were published after 2000, followed by the 1990s, and the 1980s. The total sample size for all the articles included in our review was 13,379 patients. Two-thirds of the trials were single-center RCTs and four-fifths of the trials were conducted in Europe. 42 of 70 trials assessed management of femoral neck fractures whereas the remaining 28 assessed patients with intertrochanteric fractures. Overall, 38 studies compared different methods of internal fixation, 14 compared arthroplasty with internal fixation, and 14 others compared methods of arthroplasty (Table 1).

**Table 1.** Study characteristics

Characteristics	Number of studies
Publication year	
1980–1989	8
1990–1999	22
2000–present	40
Geographic location	
Europe	58
North America	7
Asia	5
Sample size	
< 99	14
100–199	24
200-499	30
> 500	2
Number of centers	
1	50
2–4	8
> 5	4
NR	8
Average patient age	
60–69	2
70–79	22
80–89	43
NR	3
Type of fracture	
Intertrochanteric	28
Femoral neck	42
Intervention	
Arthroplasty vs. IF	14
THA vs. IF	4
HA vs. IF	10
THA vs. HA vs. IF	3
Arthroplasty	14
THA vs. THA	2
THA vs. HA	5
HA vs. HA	7
IF vs. IF	38
IF vs. EF	1

NR: not reported; EF: external fixation; IF: internal fixation; THA: total hip arthroplasty; HA: hemiarthroplasty.

Study characteristics were also analyzed based on type of fracture and, subsequently, year of publication. Age and sample size were similar in all time periods in studies evaluating both intertrochanteric and femoral neck fractures (Tables 2 and 3). For intertrochanteric fractures, internal fixation was the most commonly evaluated intervention in all time periods (Table 2). With respect to femoral neck fractures, internal fixation was the intervention most commonly studied during the 1990s, while at least 1 study arm evaluating arthroplasty has been studied most since 2000 (Table 3).

**Table 2.** Study characteristics of randomized controlled trials assessing intertrochanteric fractures

	Pre-2000 (14 trials)	2000+ (14 trials)	p-value
Average age	80	80	0.8
Average sample size **^a^**	158	198	0.4
Sample size range **^a^**	80–378	58–598	
Intervention **^b^**			
Arthroplasty vs. arthroplasty	0	0	
Arthroplasty vs. IF	1	1	
IF vs. IF	13	12	
IF vs. EF	0	1	

**^a^** Patients

**^b^** IF: internal fixation; EF: external fixation.

**Table 3.** Study characteristics of randomized controlled trials assessing femoral neck fractures

	1980s (4 trials)	1990s (12 trials)	2000+ (26 trials)	p-value
Average age	77	79	81	0.2
Average sample size **^a^**	201	218	192	0.8
Sample size range **^a^**	89–278	43–607	40–455	
Intervention **^b^**				
Arthroplasty vs. arthroplasty	1	2	11	
Arthroplasty vs. IF	2	2	11	
IF vs. IF	1	8	4	

**^a^** Patients

**^b^** IF: internal fixation.

### Mortality over time

**Figure 1:**
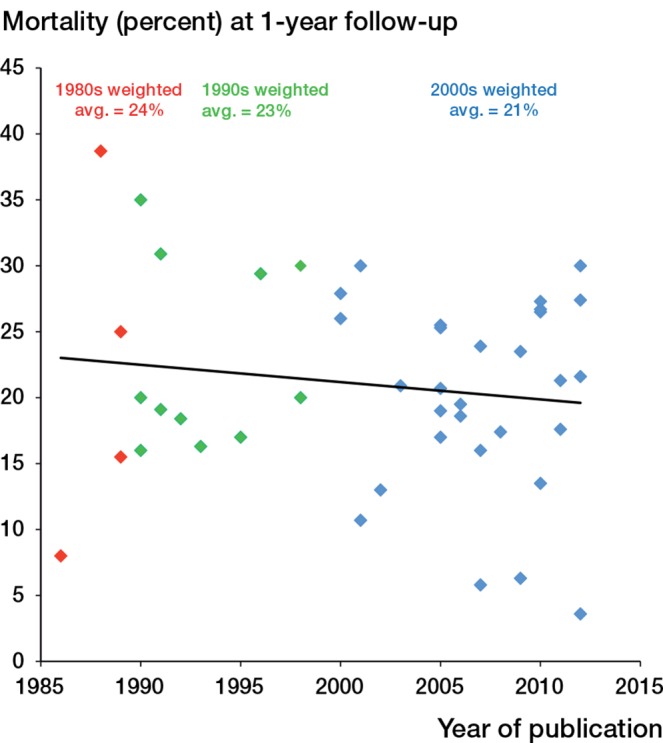
Mortality rate 1 year postoperatively for studies published in the 1980s, 1990s, and 2000s. Average 1-year mortality for each time period was calculated using the mortality rate of each trial in that time frame, weighted by sample size.

**Figure 2.**
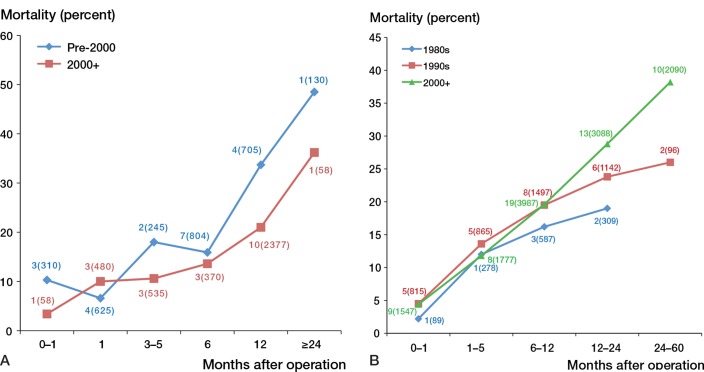
Mortality rates by fracture type. A. Mortality rates in RCTs assessing treatment of intertrochanteric fractures: mean rates for trials published before 2000 and after 1999. B. Mortality rates in RCTs assessing treatment of femoral neck fractures: mean rates for trials published in the 1980s, 1990s, and 2000s. Number of trials and combined sample size of these trials (in brackets) are given next to each data point.

44 studies evaluated one-year mortality rates. Weighted average mortality rates 1 year postoperatively changed from 24% (95% CI: 5–44) for RCTs in the 1980s to 23% (CI: 19–28) in the 1990s, and to 21% (CI: 19–24) after 1999. Despite an overall decrease in mortality over time (Figure 1), the differences in mean mortality rates were not statistically significant (p = 0.7). Analysis by fracture type revealed a reduction in the weighted 1-year mortality rate for intertrochanteric fractures; the mean mortality decreased from 34% to 23% in RCTs published before the year 2000 and in the years after 1999 (p = 0.005). Conversely, the weighted mean 1-year mortality rates for femoral neck fractures increased from 19% in the 1980s to 20% in the 1990s, and to 20% after 1999 (p = 0.8) (Figure 2).

### Reoperation rate over time

19 studies evaluated reoperation rates 1 year postoperatively, and 44 measured mortality. The weighted mean reoperation rate 1 year postoperatively changed from 9.9% for RCTs in the 1980s to 11% in the 1990s, and to 6.6% after 1999 (p = 0.6) (Table 4).

**Table 4.** Mortality and reoperation rates in femoral neck and intertrochanteric hip fractures over time

	No. of RCTs	1980s	1990s	2000+	p-value
Mortality rate **^a^**					
Overall	44	24%	23%	21%	0.7
Intertrochanteric	14	34%		23%	0.005 **^b^**
Neck	30	19%	20%	20%	0.8
Reoperation rate **^a^**					
Overall	19	9.9%	11%	6.6%	0.6

**^a^** Mortality and reoperation rates are weighted by sample size.

**^b^** Statistically significant.

## Discussion

Over the past 3 decades, after surgical interventions in hip fracture patients there was no statistically significant reduction in 1-year mortality rates. An exception to this was the mortality rates for intertrochanteric fractures, which showed a statistically significant reduction in mean 1-year mortality rate of 11%. Despite this, mean mortality for intertrochanteric fractures since the end of 1999 has remained at 23%. In total, 4 trials found a 1-year mortality of less than 10%, 3 of which were published after the year 2000. Importantly, exclusion of these outlier trials in a sensitivity analysis did not influence the statistical significance of our results. We have also demonstrated an overall decreasing—but not statistically significant—reoperation rate 1 year postoperatively for all hip fracture patients.

Few studies have analyzed trends in mortality rates following hip fractures, and among these there have been conflicting findings (Haleem et al. 2008, Brauer et al. 2009). Using a sample of the United States Medicare population, Brauer et al. conducted a retrospective observational study of 786,717 hip fracture patients between 1986 and 2005. In an age- and risk-adjusted analysis, they found a statistically significant reduction in 1-year mortality rates for both men and women over the 20-year period. Specifically, mortality rates decreased from 41% to 33% (Relative Risk Reduction (RRR) = 20%, p < 0.001) in men and from 24% to 22% (RRR = 8.8%, p < 0.001) in women. Although these findings reached statistical significance owing to the impressively large sample size of patients, the absolute risk reduction was only 2% in females, which is comparable to our findings of overall mortality. This trial covered a large cohort of patients, but the generalizability may be limited as it was based solely in the United States (Brauer et al. 2009). Haleem et al. systematically reviewed mortality rates in 36 studies published between 1959 and 1998, which showed no trend in 1-year mortality over 4 decades. Specifically, the mortality rates in the 4 decades from the 1960s to the 1990s were 27%, 31%, 22%, and 22%. The authors concluded that there was no appreciable change in mortality rates over time (Haleem et al. 2008). The present study differs from this review, as we exclusively reviewed randomized controlled trials that collected data prospectively. Furthermore, our review included studies published until 2012, which represents an additional 14 years, and 29 RCTs that evaluated 1-year mortality. Despite having differences in methodologies and in findings of statistical significance, all 3 studies (including ours) found recent 1-year mortality rates of almost 20% with marginal decreases over time. The exception was the male population in the study by Brauer et al., which had a mortality rate of 33%.

It is well documented that certain patient characteristics, such as age and cognitive status, substantially influence post-fracture outcomes. Specifically, advanced age and cognitive impairment are both associated with an increase in mortality after hip fracture (Johansson et al. 2000, Bottle and Aylin 2006). However, in the trials included here the average values for age were similar over the different time periods. Furthermore, there is no reason to believe that there is any disparity in the proportion of patients suffering from cognitive impairment who are included in hip fracture trials over time. It is unlikely that our results were biased by patient characteristics, in showing an improvement in mortality for intertrochanteric hip fractures over time or lack of such improvement for femoral neck fractures.

The one characteristic identified among studies evaluating femoral neck fractures was a shift from assessing internal fixation techniques in the 1990s to the majority of studies evaluating arthroplasty in the 2000s. However, the mortality rates were similar (∼20%) in these 2 eras. This finding remains consistent with current evidence. In a recently published systematic review evaluating the effect of surgical procedures on outcomes after hip fracture, an analysis of 33 randomized trials on femoral neck fractures showed no difference in mortality rates based on operative intervention. This included comparison of various methods of internal fixation, comparison of internal fixation and arthroplasty (hemi and total), and comparison of hemiarthroplasty and total hip arthroplasty (Butler et al. 2011). That review also covered 40 randomized trials evaluating intertrochanteric fractures and found no differences in mortality rates between plate-and-screw devices and intramedullary nails (compared amongst or against each other).

With 1-year mortality rates still around 20%, this highlights the need to improve outcomes. New research has identified that mortality and reoperation rates can be improved by optimizing perioperative care and surgical techniques. In a meta-analysis of 13,478 patients in 16 observational studies, Simunovic et al. (2010) found a statistically significant reduction in mortality in patients who underwent hip fracture surgery within 1–3 days of injury. Specifically, the risk of mortality at 1 year postoperatively was reduced by 45%. Postoperative care pathways have also proven to be pivotal in reducing mortality rates. In a randomized trial of 319 patients admitted to a Spanish university hospital for hip fracture surgery, the patients were either randomized to a multidisciplinary geriatric intervention group or to conventional (usual) care. Those who were randomized to the intervention group had a statistically significantly lower in-hospital mortality rate (0.6%) than the usual care group (6%) (Vidan et al. 2005).

Furthermore, we can also look to improve the methods of surgical treatment for hip fractures to improve reoperation rates. Management of displaced femoral neck fractures by means of internal fixation has been shown to carry a reoperation risk of greater than 6 times the risk associated with hemiarthroplasty. Such risk is mainly attributable to fracture non-union and often occurs within 1 year (Murphy et al. 2013). Operative techniques should be further optimized as well. For instance, in a recently published review it has been demonstrated that tip-to-apex distance of the lag screw during internal fixation should be less than 25 mm, as higher values are associated with a statistically significant increase in cutout failure (Rubio-Avila et al. 2013). As indicated, early surgery, optimal care pathways, and optimal decision making regarding surgery can further improve patient outcomes after hip fractures.

The present study has several strengths. Using a systematic approach, we were able to collect data from 70 RCTs, which were published over a long time period. The RCTs were conducted in various geographic locations, using a variety of surgical interventions. This increased the generalizability of our findings. A limitation of this review was comprehensive access to data, as we were unable to obtain 8 articles through our institution, which could have provided additional results. Furthermore, there were few relevant RCTs published before 1990, as only 8 trials were included from the 1980s. Accordingly, our mean mortality rates for studies in the 1980s were based on a smaller sample size. Nevertheless, we maintained a minimum cutoff of 3 primary mortality rates for calculation of mean. Lastly, our systematic review focused exclusively on randomized trials, which may have resulted in a selection bias, as these studies often under-represent cognitively impaired and nursing home patients. As these patients often have poorer outcomes, the mean mortality calculated for each era may have been an underestimate of the true mortality. However, this limitation probably did not alter our primary outcome of change in mortality over time.
